# Supplementary material for: Effectiveness of Antimicrobial Lock Therapy for the Treatment of Catheter-Related and Central-Line-Associated Bloodstream Infections in Children: A Single Center Retrospective Study
Source: Antibiotics (Basel). 2023 Apr 23;12(5):800. doi: 10.3390/antibiotics12050800 (PMC10215690; doi:10.3390/antibiotics12050800)
Supplement: Supplementary file 1 [file antibiotics-12-00800-s001.zip › antibiotics-2330050-supplementary.pdf]

Table S1. Univariate analysis for ALT risk factors.

	ALT Success (n/N)	ALT Success (%)	Odds Ratio	95% Confidence Interval	*p*-Value
**Age class (months)**					
≤ 60	12/19	63.1	0.65935	0.16419-2.6478	0.5571
>60	13/18	72.2	1.5166	0.37767-6.0905	
**Gender**					
Male	16/25	64	0.59262	0.12693- 2.767	0.5057
Female	9/12	75	1.6874	0.36141-7.8786	
**ANC (cell/mm3)**					
≤ 5000	15/24	62.5	0.50002	0.10804-2.3141	0.3753
>5000	10/13	76.9	1.9999	0.43213-9.2556	
**CPR (mg/dl)**					
≤5	15/20	75	2.0999	0.51833-8.5075	0.2986
>5	10/17	58.8	0.47621	0.11754-1.9293	
**Procalcitonin (ng/ml) ***					
≤5	9/15	60	0.577	0.134-2.48	0.4602
>5	13/18	72.2	1.73	0.403, 7.46	
**Type of infection**					
CRBSI	6/10	60	0.6316	0.13939-2.8618	0.5511
CLABSI	19/27	70.4	1.5833	0.34943-7.174	
**Catheter-days****					
≤150	8/11	72.7	1.3334	0.276-6.4415	0.7204
>150	16/24	66.7	0.74999	0.15524-3.6232	
**MDR*****					
Yes	8/14	57.1	0.33334	0.07265-1.5295	0.1575
No	16/20	80	3	0.65383-13.7647	
**Time to ALT, (days)**					
≤5	16/21	76.2	2.4888	0.60844-10.1803	0.2045
>5	9/16	56.2	0.4018	0.098229-1.6435	
**Taurolidine**					
Yes	1/4	25	0.12501	0.011461-1.3635	0.0881
No	24/33	72.7	7.9997	0.73342-87.2551	

**CVC insertion site infection**<br>Yes<br>No	 3/5<br>22/32	 60<br>68.7	 0.6818<br>1.467	 0.09807-4.74<br>0.211- 10.196	0.6987
**Dwell time of 24 h**<br>Yes<br>No	 22/30<br>3/7	 73.3<br>42.8	 3.6667<br>0.27272	 0.66873-20.1051<br>0.049739-1.4954	0.1345

* Total of 33 episodes, excluding missing data; ** Total of 35 episodes, excluding missing data; ***Total of 34 episodes, excluding fungi.
